# Feeding sunflower meal with pullets and laying hens even at a 30% inclusion rate does not impair the ileal digestibility of most amino acids

**DOI:** 10.3389/fvets.2024.1347374

**Published:** 2024-01-25

**Authors:** Nikoletta Such, Ákos Mezőlaki, Kesete Goitom Tewelde, László Pál, Boglárka Horváth, Judit Poór, Károly Dublecz

**Affiliations:** ^1^Department of Nutrition and Nutritional Physiology, Institute of Physiology and Nutrition, Hungarian University and Agriculture and Life Sciences, Keszthely, Hungary; ^2^Agrofeed Ltd., Győr, Hungary; ^3^Department of Animal Sciences, Hamelmalo Agricultural College, National Higher Education and Research Institute, Keren, Eritrea; ^4^Institute of Mathematics and Basics of Natural Sciences, Hungarian University of Agriculture and Life Sciences, Keszthely, Hungary

**Keywords:** sunflower meal, laying hens, pullet, amino acid, digestibility

## Abstract

The use of locally available protein sources in poultry nutrition is challenging for feed manufacturers and farmers. Sunflower meal (SFM) is available in high quantities in several European countries and could be used as a poultry feedstuff at higher inclusion rates. However, its maximum inclusion rate in the diets of different poultry species and age categories is unknown. Pullets and laying hens can probably tolerate higher amounts of SFM, but only limited information is available on these poultry groups. Therefore, a digestibility trial was carried out with 8-week-old layer type pullets and 50-week-old laying hens. Beside a basal diet, SFM was fed at 10, 20 and 30% inclusion rates. Feeding SFM significantly improved the digestibility of essential amino acids (AA) of threonine, valine, lysine, tyrosine, glycine, aspartic acid, and arginine in the pullet diets. No such improvement was found in laying hens. Only the absorption of the two branch-chain AAs, leucin (pullets) and isoleucine (hens), declined due to SFM. The AA digestibility of the SFM itself was also calculated by linear regression. The coefficients were, in all cases, higher in hens than in pullets. Comparing the measured digestibility coefficients of SFM with table values, it can be concluded that high variance exists because of the differences in the methodology and the test animals in the digestibility trials. From the present trial, it can be concluded that SFM can entirely replace extracted soybean meal in pullet and layer diets, without negative effects on the protein digestion of birds.

## 1 Introduction

Protein is one of the most expensive components of animal diets and its amount is increasingly limited around the world (1). Soybean meal is the dominating protein source for farm animals in Europe. Because the cultivation of soybean is focused mostly in America, its transportation around the world has a high environmental impact (2–5). Therefore, the importance of locally available protein sources, legume seeds, and industrial by-products will increase in the future (6).

Sunflower is a widely cultivated crop, the third biggest in global oil seed production (7, 8). Across the EU in 2021, the harvested production of sunflower seed was 10.4 million tons. Sunflower meal (SFM) is a byproduct of the oil industry and it can be used as an alternative protein source in farm animal nutrition (2, 3, 5, 9). The crude protein content of SFM shows high variance (23–44%), depending mainly on the quality of the dehulling procedure. The use of SFM in poultry diets is limited due to its high fiber and low energy content, its low concentration of lysine (LYS) and threonine (THR), and the presence of different polyphenolic compounds (10, 11).

Sunflower contains a very diverse fiber composition, including both structural and water-soluble fractions. Its structural, insoluble fiber, which can be found mainly in the hulls, can stimulate gizzard development and by this process, may increase the retention time of the digesta in the upper part of the GIT. Proper gizzard function also stimulates pancreatic enzyme secretion, improving the digestibility of starch, lipids, and other dietary components on the GIT (12). In the water-soluble fraction, β-glucans dominate. SFM’s β-glucan, like the β-glucans found in cereals, can increase the viscosity of the gut content, which is associated with reduced nutrient absorption and imbalance of the microbiota in the small intestine (13, 14). For this reason, NSP-degrading enzymes are used also if SFM-containing diets are provided (15). The positive effect of this addition on nutrient utilization and production traits has already been demonstrated by numerous studies (12). Our knowledge of the specific effects of SFM’s fiber on the digestion and gut health of birds is incomplete, and we do not know its maximal inclusion rates for the different poultry species and age categories (16). Using the last generation exogenous enzymes, we can also modify the negative effects of the different fiber fractions. Its considerably high fiber limits its use in broilers (8, 12, 17–19). However, according to several studies, SFM can be utilized in the diets of laying hens with no negative impact on egg quality parameters (9, 20, 21). This can be explained by the fact that layers have a more developed digestive system in terms of gut capacity compared to broilers. Laying hens have a lower protein requirement than broiler chickens, which makes it possible to replace soybean meal completely with SFM (22). In the case of pullets, the use of insoluble fiber has been shown to be beneficial for the development of the gastrointestinal tract (GIT) (23, 24). In the study of Abdallah and Beshara (23), supplementing the pullets’ diet with 7 and 14% sunflower meal from 11 to 19 weeks resulted in significantly improved live weight and FCR compared to the SFM-free control diet. The protein evaluation of poultry feedstuffs is based on the so-called standardized ileal amino acid digestibility (SID). The determination of SID is based on the evaluation of the AA content of the whole or terminal ileum content, assuming that the amino acids of this gut segment are not digestible (25). This term is used to express the amino acid content of the feeds and the requirements of the birds. Rodehutscord et al. (26) developed a linear regression method as a tool to study the AA digestibility of raw materials in chickens. In this case, the test feedstuff is incorporated into the test diets at the expense of starch at graded levels. The increased protein content of the diets and the AA intake of animals is related only to the test feedstuff. Therefore, the slope of the linear regression between the AA intake and pre-cecally absorbed AA content means the digestibility of the AAs. A further advantage of this method is that it can also give information on the maximal inclusion rate of the feedstuffs without impairing digestion. In the present work, this method was used for AA digestibility determination.

Most of the animal experiments on SID measurements have been carried out in broiler chickens and limited research data are available regarding pullets and laying hens (25, 27). Pullets are reared with a restricted feeding and light program to achieve the optimal live weight at the start of the laying period. The low amount of daily feed intake is an important difference between broiler chickens and pullets, which could affect protein digestibility. In the case of laying hens, the protein, energy, and calcium requirements change during the day due to the synthesis of egg components, which modify the feeding habits of hens. The longer dark period and the restricted feeding means also difference from broiler chickens (28, 29). According to the current research intended to assess the effect of dietary inclusion of SFM as a complementary protein resource at 10, 20, and 30% on the ileal amino acid digestion of pullets and laying hens. According to the knowledge of the authors, no ileal amino acid digestibility result of SFM is available for pullets and layers. The measured values have been compared with table values.

## 2 Materials and methods

The trials were carried out at the experimental farm of the Institute of Physiology and Nutrition, Hungarian University of Agriculture and Life Sciences (Georgikon Campus, Keszthely, Hungary). The animal experiments were approved by the Institutional Ethics Committee (Animal Welfare Committee, Georgikon Campus, Hungarian University of Agriculture and Life Sciences) with the number MÁB-11/2019.

### 2.1 Experiment 1

In the first experiment, a total of 32 Tetra SL pullets were individually housed in metabolic cages. The special feeders made possible the exact measurement of daily feed intakes. The water was available *ad libitum* through nipple drinkers. In the beginning, the pullets were 10 weeks old with an average body weight of 638 g. Alongside a corn, wheat, and cornstarch-based control diet (C), three diets containing graded levels of SFM were used. The proportions of SFM were 10, 20, and 30% (SFM10, SFM20, SFM30). All diets were fed in 8 replicate pullets. Sunflower meal was fed at the expense of wheat starch, and consequently, the increase in the AA concentrations of the experimental diets originated from SFM only. Titanium dioxide (TiO_2_) was used as an indigestible marker at 0.5%. The nutrient content of SFM can be found in Table 1, while the composition and nutrient content of the experimental diets are shown in Table 2. The AMEn content of SFM and the diets were calculated with the equation of McNab and Fisher (31). As can be observed, the increased SFM incorporation increased both the crude protein and crude fiber contents of the diets. All diets were fed in mash form and the daily feed intake was adjusted to the breeder’s nutritional guide (32). The length of the light and dark periods was 10 and 14 h, respectively. Computer-controlled climatic conditions were maintained during the trial according to the breeder’s recommendations (33).

**Table 1:** Nutrient content of the sunflower meal (g/kg).

Nutrient content of SFM	
Dry matter	920.8
Crude protein	349.5
Crude fat	8.0
Crude fiber	184.8
Ash	71.3
Predicted AMEn (MJ/kg)*	6.61
Amino acid content of SFM	
Cystine	6.0
Aspartic acid	33.8
Methionine	8.5
Threonine	13.7
Serine	15.7
Glutamic acid	72.9
Proline	15.0
Glycine	21.0
Alanine	15.9
Valine	18.2
Isoleucine	14.6
Leucine	21.9
Tyrosine	8.3
Phenylalanine	16.9
Histidine	9.3
Lysine	12.7
Arginine	31.8

*The predicted AMEn content of sunflower meal was calculated with the equation of the European Table of Energy Values for Poultry Feedstuffs (30).

**Table 2:** Composition and measured nutrient contents of pullet diets (g/kg).

	C	SFM10	SFM20	SFM30
Composition of experimental diets				
Maize	415	415	415	415
Starch	300	200	100	0
Wheat	200	200	200	200
Ext. sunflower meal	0	100	200	300
Sunflower oil	50	50	50	50
Limestone	14	14	14	14
MCP^1^	7	7	7	7
Premix^2^	5	5	5	5
NaCl	3	3	3	3
NaHCO_3_	1	1	1	1
TiO_2_	5	5	5	5
	1,000	1,000	1,000	1,000
Measured nutrient contents				
Dry matter	891.7	891.7	891.9	892.4
Crude protein	53.0	84.5	118.2	146.6
Crude fat	67.3	68.2	73.8	72.9
Crude fiber	17.8	33.7	48.4	64.6
Ash	39.7	45.2	52.4	56.5
Ca	8.3	8.9	8.8	8.9
Predicted AMEn (MJ/kg)*	13.96	13.57	13.05	12.28
Cystine	1.2	1.7	2.3	2.8
Aspartic acid	3.2	6.2	9.4	12.2
Methionine	1.0	1.7	2.5	3.2
Threonine	1.8	3.0	4.3	5.4
Serine	2.5	3.9	5.4	6.7
Glutamic acid	12.4	18.8	25.8	31.6
Proline	5.0	6.4	8.0	9.2
Glycine	2.1	4.1	6.2	7.9
Alanine	3.0	4.4	6.0	7.2
Valine	2.4	4.0	5.8	7.3
Isoleucine	1.8	3.2	4.6	5.8
Leucine	5.1	7.2	9.5	11.3
Tyrosine	1.4	2.3	3.1	3.9
Phenylalanine	2.5	4.1	5.7	7.1
Histidine	1.4	2.2	3.1	3.9
Lysine	1.6	2.7	4.0	5.1
Arginine	2.6	5.3	8.2	10.7

C, control; SFM10, control diet supplemented with 10% extracted sunflower meal; SFM20, control diet supplemented with 20% extracted sunflower meal; SFM30, control diet supplemented with 30% extracted sunflower meal. ^1^MCP, monocalcium phosphate. ^2^Pullet premix was supplied by Agrofeed Ltd. (Győr, Hungary). The active ingredients contained in the premix were as follows (per kg of diet): vitamin A—2,000,000 NE, vitamin D3—600,000 NE, vitamin E—5,000 mg, menadione—450 mg, thiamine—450 mg, riboflavin—1,320 mg, pyridoxin HCl—720 mg, cyanocobalamin—4 mg, niacin—6,000 mg, pantothenic acid—1,680 mg, folic acid—216 mg, biotin—20 mg, betaine—14,060 mg, BHT—75 mg, BHA—75 mg, citric acid—67.5 mg, Zn (as ZnO)—14,000 mg, Cu (as CuSO_4_5H_2_O)—1,600 mg, Fe (as FeSO_4_H2O)—6,000 mg, Mn (as MnO)—20.000 mg, I [as Ca(IO_3_)_2_]—200 mg, Se (as Na_2_SeO_3_)—60 mg, endo-1.4-beta-xylanase—244,000 U, Endo-1.3(4)-beta-glucanase—30,400 U, 6-phytase—100,000 FTU. *The AMEn content of diets was calculated with the equation of McNab and Fisher (31).

### 2.2 Experiment 2

In the second trial, a total of 32 Teta SL laying hens were used and housed in the same metabolic cages as described in the first experiment. At the beginning of the experiment, the hens were 50 weeks old, with an average body weight of 1,941 g. The composition and nutrient content of the hen diets are shown in Table 3. The lengths of the light and dark periods were in this case 16 and 8 h, respectively. All the housing and experimental conditions were the same as described in the first experiment.

**Table 3:** Composition and measured nutrient contents of layer diets (g/kg).

	C	SFM10	SFM20	SFM30
Composition of experimental diets				
Maize	331	331	331	331
Starch	300	200	100	0
Wheat	200	200	200	200
Extr. sunflower meal	0	100	200	300
Sunflower oil	50	50	50	50
Limestone	98	98	98	98
MCP	7	7	7	7
Premix^2^	5	5	5	5
NaCl	3	3	3	3
NaHCO_3_	1	1	1	1
TiO_2_	5	5	5	5
	1,000	1,000	1,000	1,000
Measured nutrient contents				
Dry matter	890.9	896.9	897.9	900.2
Crude protein	42.8	75.6	112.4	146.2
Crude fat	63.6	63.7	65.2	64.6
Crude fiber	16.2	35.7	59.9	76.3
Ash	121.2	123.1	127.1	133.2
Ca	51.6	48.8	48.6	4.92
Predicted AMEn (MJ/kg)*	13.10	12.31	11.51	10.73
Cystine	1.0	1.6	2.1	2.9
Aspartic acid	2.8	5.4	8.5	12.2
Methionine	0.9	1.5	2.5	3.0
Threonine	1.6	2.6	4.2	5.5
Serine	2.2	3.6	5.2	6.6
Glutamic acid	9.5	16.9	25.0	32.0
Proline	3.9	5.7	7.8	9.5
Glycine	1.8	3.5	6.0	8.0
Alanine	2.3	4.1	5.7	7.0
Valine	1.8	3.7	5.6	7.4
Isoleucine	1.6	2.9	4.3	5.9
Leucine	4.1	6.4	8.9	11.3
Tyrosine	1.3	2.1	2.9	3.8
Phenylalanine	2.1	3.5	5.3	7.2
Histidine	1.2	2.2	2.8	3.6
Lysine	1.5	2.4	3.9	5.2
Arginine	2.2	4.8	7.9	10.4

C, control; SFM10, control diet supplemented with 10% extracted sunflower meal; SFM20, control diet supplemented with 20% extracted sunflower meal; SFM30, control diet supplemented with 30% extracted sunflower meal; MCP—monocalcium phosphate; 1 Premix was supplied by Agrofeed Ltd. (Győr, Hungary). The active ingredients contained in the premix were as follows (NE per kg of diet): vitamin A—2,000,000 NE, vitamin D3—600,000 NE, vitamin E—6,000 mg, menadione—400 mg, thiamine—436 mg, riboflavin—1,200 mg, pyridoxin HCl—600 mg, cyanocobalamin—4 mg, niacin—6,254 mg, pantothenic acid—1825 mg, folic acid—300 mg, biotin—30 mg, betaine—30,000 mg, BHT—79.5 mg, BHA—79.5 mg, citric acid—71.5 mg, Zn (as ZnO)—8,000 mg, Zn (as 3b607)—8,000 mg, Cu (as 3b413)—2,000 mg, Fe (as FeSO_4_H2O)—10,000 mg, Mn (as MnO)—10,000 mg, Mn (as 3b506)—10,000 mg, I [as Ca(IO_3_)_2_]—300 mg, Se (as C_5_H_11_NO_2_Se)—40 mg, endo-1.4-beta-xylanase—244,000 U, Endo-1.3(4)-beta-glucanase—30,400 U, 6-phytase—100,000 FTU. *The AMEn content of diets was calculated with the equation of McNab and Fisher (31).

### 2.3 Sample collection

During a 5-day adaptation period, the pullets were accommodated in metabolic cages and consumed their daily rations entirely. On the 6th and 7th days, the daily feed intake of the animals was measured. On the 7th day, the birds were slaughtered by asphyxiation with carbon dioxide, and the ileal contents were collected immediately. The samples were collected from the Meckel’s diverticulum up to 1 cm before the ileocecal junction. The ileum was cut into short pieces, then the intestinal contents were pushed out gently, homogenized, and stored in Eppendorf tubes at −20°C until further analysis.

#### 2.3.1 Analysis and calculations

The proximate analysis of SFM and compound feeds was carried out with the official methods: dry matter (ISO 6496:2001), crude protein (ISO 5983-1:2005), crude fiber (ISO 6865:2001), crude fat (ISO 11085:2015), crude ash (ISO 5984:1992), and amino acids (ISO 13903:2005). Amino acid contents of feed and ileal samples were determined with an automatic amino acid analyzer (Ingos Amino Acid Analyzer AAA 400) after 24 h of acid hydrolysis with 6 M aqueous HCl at 110°C. To avoid the loss of methionine (MET) and cystine (CYS), before hydrolysis, samples were oxidized with formic acid. Tryptophan contents were not determined. The TiO_2_ content was determined by a spectrophotometer (Jenway 6100) at 410 nm, according to the method of Short et al. (34).

The apparent amino acid digestibility of the diets was calculated from the amino acid and TiO_2_ contents of feeds and ileal digesta using the following equation:


$$\begin{array}{l} {\mathrm{DC}}_{\mathrm{AA}\;\mathrm{diet}}:\\ \left(\left({\mathrm{AA}}_{\mathrm{diet}}-\left({\mathrm{AA}}_{\mathrm{digesta}}\;\;x\;{\mathrm{Tid}}_{2\;\mathrm{Diet}}/{\mathrm{TiO}}_{2\;\mathrm{digesta}}\;\right)\right)/{\mathrm{AA}}_{\mathrm{diet}}\;\right)\times 100 \end{array}$$

where:

${\mathrm{DC}}_{\mathrm{AA}\;\mathrm{diet}}\;$ = amino acid digestibility coefficient of the diets (%)

AA_ddiet_ = amino acid content of the diet (mg/g)

AA_digesta_ = amino acid content of the ileal digesta (mg/g)

TiO_2diet_ = titanium dioxide content of the diet (%)

TiO_2digesta_ = titanium dioxide content of the ileal digesta (%).

The ileal amino acid digestibility of sunflower meal was calculated by linear regression between the daily amino acid intake and the amount of the pre-cecally absorbed amino acids, as described by Rodehutscord et al. (26). The daily intake of the AAs (mg/day) was calculated by multiplying the feed intake (g/d) by the AA content of the diet (mg/g). The quantity of pre-cecally absorbed AAs was calculated as AA intake (mg/day) times the ileal amino acid digestibility of the diets (DC_AA Diet_). The AA digestibility of SFM was the slope of the linear regression equation. The measured AA digestibility of SFM was compared with those of the tables (35–37).

The AA digestibility of the diets was compared with one-way ANOVA, while the comparison of the measured AA digestibility values of SFM with those can be found in the tables was evaluated with multivariate ANOVA. The linear regression analysis was carried out using the following formula: Y_i_ = β_0_ + β_1_ × X_i_, where Y_i_ = dependent variable (ileal digested AA); β_0_ = constant; β_1_ = slope; X_i_ = independent variable (ingested AA). All the statistical analysis including the linear regression was carried out using the software package SPSS 24.0 for Windows (SPSS Inc., Chicago, IL, United States). The differences were considered significant at *p* < 0.05.

## 3 Results

The average daily feed intake of pullets in the C, SFM10, SFM20, and SFM30 groups were 53, 59, 58, and 58 g, respectively. Therefore, the birds consumed slightly more feed in the SFM-containing diets. In the case of pullets, the digestibility of the individual AAs of the four diets ranged between 58.6 and 88.9%, with the lowest and highest values being determined for threonine and glutamine, respectively (Table 4). Among the essential AAs, the digestion of MET was the highest (86.3%). Despite the higher fiber content of the SFM-containing diets, the absorption of certain amino acids was significantly increased. Among essential amino acids, the SFM significantly increased the digestibility of THR, VAL, LYS, and ARG. Leucine (LEU) was the only essential AA, of which digestibility was affected negatively. The digestibility of three non-essential amino acids, GLY, TYR, and ASP, also increased significantly.

**Table 4:** The ileal amino acid digestibility of pullet diets (%).

Essential amino acids												Non-essential amino acids					
%	MET*	THR	CYS	VAL	ILE	LEU	PHE	HIS	LYS	ARG	TYR	GLY	ASP	SER	GLU	PRO	ALA
C	84.34	58.60^b^	73.44	74.86^b^	79.34	85.00^a^	82.50	77.05	69.85^b^	80.09^b^	70.24^b^	70.23^b^	72.23^b^	72.51	88.88	81.08	78.66
SFM10	84.80	64.40^a^	73.88	76.56^ab^	79.99	82.73^ab^	82.94	77.58	73.04^ab^	82.58^ab^	76.33^a^	73.11^ab^	74.60^ab^	73.12	87.55	81.67	77.74
SFM20	86.31	66.90^a^	74.10	79.20^a^	80.44	82.48^ab^	82.96	77.52	73.20^ab^	84.49^a^	78.46^a^	74.40^a^	77.08^a^	74.88	88.02	82.37	77.99
SFM30	84.38	66.75^a^	73.91	78.31^ab^	80.33	79.67^b^	82.33	76.67	74.04^a^	85.17^a^	79.07^a^	73.32^ab^	75.18^ab^	73.26	86.39	81.24	77.19
Pooled SEM	0.004	0.007	0.004	0.005	0.005	0.005	0.004	0.005	0.005	0.050	0.007	0.005	0.006	0.005	0.004	0.004	0.004
*p*-value	0.384	**0.000**	0.972	**0.030**	0.881	**0.001**	0.957	0.929	**0.027**	**0.001**	**0.000**	**0.031**	**0.007**	0.503	0.277	0.763	0.768

^a,b^Means with different superscripts of the same column are significantly different (*p* < 0.05). *Methionine, threonine, cystine, valine, isoleucine, leucine, phenylalanine, histidine, lysine, arginine, tyrosine, glycine, aspartic acid, serine, glutamine, proline, alanine. *n* = 8 for each diet.The bold *p*-values show the significantly differences (*p*<0.05).

In the laying hen trial, in contrast with the pullets, the average daily feed intake decreased with the increased proportion of SFM (control: 117 g, SFM10: 101 g, SFM20: 86 g, and SFM30: 77 g). The digestibility interval of the AAs was between 73.6 and 93.6% (Table 5). In this case, MET was the most highly digested amino acid (90.31%). In the trial with laying hens, feeding SFM did not modify the digestibility of AAs. The only significant difference was the impaired digestibility of ILE.

**Table 5:** The ileal amino acid digestibility of layer diets (%).

Essential amino acids												Non-essential amino acids					
%	MET*	THR	CYS	VAL	ILE	LEU	PHE	HIS	LYS	ARG	TYR	GLY	ASP	SER	GLU	PRO	ALA
C	90.31	73.55	81.65	84.94	88.35^ab^	88.10	88.77	87.38	81.49	88.45	80.83	80.16	83.00	82.35	93.58	88.23	86.78
SFM10	90.09	78.57	85.82	87.02	89.25^a^	89.43	90.13	86.71	84.79	91.41	86.85	82.73	85.81	84.16	92.56	89.20	87.67
SFM20	90.07	73.69	81.97	83.73	82.70^b^	84.77	85.84	82.25	79.00	86.74	81.59	79.46	80.22	76.89	90.19	85.34	82.71
SFM30	87.77	74.53	83.04	83.13	84.68^ab^	86.32	87.75	82.95	80.29	89.64	86.01	79.55	82.42	81.32	90.44	85.30	82.88
Pooled SEM	0.006	0.017	0.005	0.011	0.009	0.009	0.007	0.009	0.014	0.008	0.013	0.013	0.004	0.012	0.005	0.007	0.009
*p*-value	0.539	0.737	0.581	0.693	**0.025**	0.363	0.250	0.094	0.450	0.298	0.296	0.875	0.519	0.214	0.087	0.154	0.098

^a,b^Means with different superscripts of the same column are significantly different (*p* < 0.05). *Methionine, threonine, cystine, valine, isoleucine, leucine, phenylalanine, histidine, lysine, arginine, tyrosine, glycine, aspartic acid, serine, glutamine, proline, alanine. *n* = 8 for each diet.The bold *p*-values show the significantly differences (*p*<0.05).

**Figure 1:**
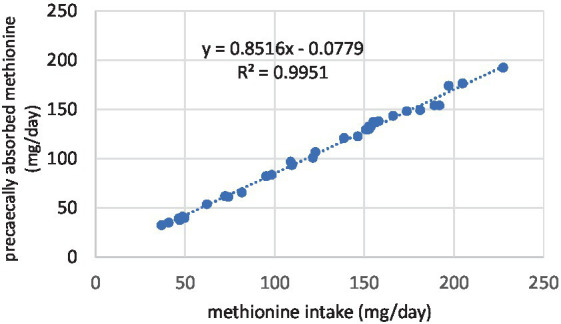
Relationship between the daily intake and ileal absorption of methionine, determined with pullets.

**Figure 2:**
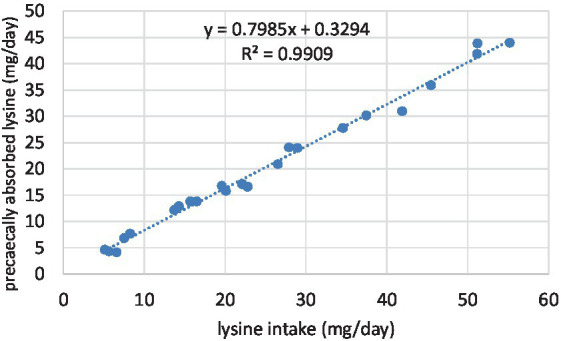
Relationship between the daily lysine intake and ileal absorption, determined with laying hens.

The details of the regression analyses are presented in Table 6. All the linear regression between the daily amino acid intake and the amount of pre-cecally absorbed amino acids were significant, with high r^2^ values. It means that feeding SFM even at 30% did not cause a decrease in protein digestion. The table shows the slopes, the constants, and the coefficients of determination. In this methodology, the slopes mean the digestibility of SFM amino acids. As indicated, the slopes of the regression lines in the pullet trial ranged between 0.70 (THR) and 0.86 (ARG, GLU). In laying hens, the lowest slope belonged also to THR (0.74), while the highest belonged to MET and ARG (0.89). For all amino acids, higher slopes were obtained in hens than in pullets. The difference between the two animal groups was small for TYR (1.4%), GLU (2.0%), PRO (2.2%), and VAL (2.9) and high for CYS (9.1%) and LEU (8.8%). Two examples of the linear regression responses are shown in Figures 1, 2.

**Table 6:** Linear regression equation parameters and their SE of estimates, describing the response of daily digested amino acids up to the terminal ileum (y) depending on the respective daily amino acid intake (x).

	Pullets			Laying hens		
	Slope	Constant	*r* ^2^	Slope	Constant	*r* ^2^
Cystine	0.737 ± 0.015	0.2 ± 1.9	0.987	0.828 ± 0.011	0.1 ± 0.2	0.996
Aspartic acid	0.766 ± 0.011	−5.8 ± 5.4	0.994	0.821 ± 0.012	0.4 ± 0.8	0.995
Methionine	0.851 ± 0.011	−0.1 ± 1.4	0.995	0.890 ± 0.010	0.1 ± 0.2	0.997
Threonine	0.700 ± 0.013	−9.9 ± 3.0	0.989	0.748 ± 0.017	0.2 ± 0.5	0.988
Serine	0.750 ± 0.015	−3.6 ± 4.3	0.989	0.804 ± 0.017	0.2 ± 0.7	0.990
Glutamic acid	0.864 ± 0.012	13.6 ± 17.4	0.994	0.885 ± 0.010	3.7 ± 1.8	0.997
Proline	0.829 ± 0.016	−5.2 ± 7.0	0.989	0.852 ± 0.010	0.9 ± 0.5	0.997
Glycine	0.744 ± 0.011	−3.4 ± 3.5	0.994	0.795 ± 0.017	0.4 ± 0.8	0.990
Alanine	0.766 ± 0.014	3.5 ± 4.4	0.991	0.820 ± 0.014	0.8 ± 0.6	0.994
Valine	0.805 ± 0.013	−7.9 ± 4.0	0.992	0.835 ± 0.015	0.4 ± 0.6	0.993
Isoleucine	0.809 ± 0.012	−1.6 ± 2.9	0.994	0.847 ± 0.013	0.2 ± 0.4	0.995
Leucine	0.775 ± 0.014	21.2 ± 7.2	0.991	0.864 ± 0.015	0.3 ± 1.0	0.994
Tyrosine	0.830 ± 0.014	−9.2 ± 2.4	0.991	0.844 ± 0.014	0.1 ± 0.3	0.994
Phenylalanine	0.822 ± 0.012	1.1 ± 3.7	0.993	0.862 ± 0.010	0.5 ± 0.4	0.997
Histidine	0.773 ± 0.014	−0.2 ± 2.3	0.991	0.829 ± 0.015	0.3 ± 0.3	0.993
Lysine	0.750 ± 0.011	−4.0 ± 2.4	0.993	0.799 ± 0.016	0.3 ± 0.5	0.991
Arginine	0.861 ± 0.009	−8.4 ± 3.7	0.997	0.892 ± 0.009	−0.0 ± 0.5	0.998

*n* = 24 for each regression line.

By comparing our results with some frequently used table values (35–37), it can be concluded that the digestibility coefficients of this trial are closer than those of CVB but show amino acid-dependent differences with the coefficients of NRC or EVONIK (Tables 7, 8). In Table 7, LYS and HIS showed the highest variance. The AA digestibility of SFM determined with pullets was below the table values in all cases except cystine. Comparing the measured and table values with multivariate ANOVA, the highest similarity was found between the EVONIK and NRC coefficients, without significant difference (*p* = 0.574). Regarding the measured coefficients, the hen digestibility values were close to those of CVB values (*p* = 0.458). The AA digestibility of pullets was significantly different from all the other groups.

**Table 7:** Comparison of the measured amino acid digestibility values of sunflower meal with table values.

	Table values			Measured values	
	Evonik (2017)	CVB (2017)	NRC (1994)	Pullet	Laying hen
Lysine	0.87	0.82	0.84	0.75	0.80
Methionine	0.92	0.92	0.93	0.85	0.89
Cystine	0.80	0.73	0.78	0.74	0.83
Threonine	0.82	0.76	0.85	0.70	0.75
Arginine	0.93	0.91	0.93	0.86	0.89
Isoleucine	0.89	0.85	0.90	0.81	0.85
Leucine	0.88	0.84	0.91	0.78	0.86
Valine	0.87	0.83	0.86	0.81	0.84
Histidine	0.88	0.77	0.87	0.77	0.83
Phenylalanine	0.90	0.87	0.93	0.82	0.86

**Table 8:** Paired comparisons of the amino acid digestibility values of sunflower meal.

		Evonik (2017)	CVB (2017)	NRC (1994)	Pullets	Hens
Evonik (2017)	*p*-value					
CVB (2017)	*p*-value	<0.001				
NRC (1994)	*p*-value	0.574	<0.001			
Pullets	*p*-value	<0.001	0.001	<0.001		
Hens	*p*-value	0.003	0.458	0.007	<0.001	

## 4 Discussion

SFM is an alternative protein source for poultry (17). However, its high fiber and low energy content, in addition to the variation in its chemical composition are the main restricting factors to its use at higher incorporation rates (11). It has been previously hypothesized that higher proportions of dietary fiber in poultry diets have a diluting effect, which was believed to cause poor nutrient utilization (38). However, the poultry industry has recognized recently that certain types and amounts of fiber could be beneficial to gastrointestinal tract development, digestion, and gut health (39). The inclusion of additional dietary fiber could also be a strategy that supports multiple aspects of laying production (40). However, according to the available results, an increase in endogenous protein and amino acid losses is inevitable if high-fiber diets are fed (41). The age of birds can also modify the endogenous amino acid losses. Higher values have been recorded in early ages because of the incomplete development of the gastrointestinal tract and lower digestion (42, 43). It was reported that the inclusion of 8% cellulose in broiler diets resulted in higher crude protein and amino acid losses (i.e., GLU, ASP, and THR) compared to diets fed with 3% cellulose. These endogenous losses might not belong to the so-called diet-specific endogenous losses (13).

Since SFM is mainly a protein source, its effect on the amino acid digestibility of the compound diets is especially important. The digestibility of amino acids in birds can be determined by different methods. The so-called difference method is most common when the test material is incorporated into a basal diet and the amino acid digestibility of the test product is calculated from the AA digestibility of the basal and test product-containing diets. The disadvantage of this method is that, if the incorporation rate of the feedstuff is low, the inaccuracy of the measurement increases. Furthermore, in this case, it is not possible to evaluate the potential depressive incorporation rates. The advantage of the regression approach is that with this method, the endogenous AA losses can also be determined (44).

The amino acid digestibility of sunflower meal was investigated in only a few cases using regression analysis (45). In this trial, SFM was fed at 15 and 30% with unsexed Ross 308 broilers until day 21. According to the results of Alagawany et al. (46), the application of a higher amount of SFM will alter the amino acid profile and crude fiber and energy content of poultry diets. Based on their results, SFM could be an acceptable feed component of poultry rations and can be fed at 25% in broiler diets and 20% in layer diets. Green et al. (47) reported that the true digestibility of essential amino acids of SFM was lower than that of soybean meal. According to our results, sunflower meal did not have a depressive effect on the amino acid digestibility of the experimental diets, even at a 30% inclusion rate. Surprisingly, the digestibility of several essential amino acids improved significantly in pullets when the SFM-containing diets were fed. These amino acids were threonine, glycine, valine, lysine, arginine, tyrosine, and aspartic acid. The only exception was leucine, of which digestibility impaired in the SFM diets. Lysine is the first limiting amino acid of SFM protein, followed by methionine, cystine, and tyrosine (46). Although glycine has been categorized as a nonessential amino acid, it may also be limiting if low-protein diets are fed (48, 49). Therefore, the improvement of glycine digestibility could be a positive result since glycine supplementation in crystalline form is not permitted in the European Union. The improvement of amino acid digestibility is in line with the results of Yokhana et al. (50). In their experiment, the dietary insoluble fiber significantly improved the digestive tract weights and the trypsin activity in the small intestine of pullets, which may contribute to an improvement in feed utilization. During their experiment, 8-week-old pullets were also used, but in contrast to our experiment, only 1% structural fiber (Arbocell RC) supplementation was used. In our study, the range of crude fiber concentration of the experimental diets was 1.78–6.46%. Similar to other findings, in this range, the crude fiber could improve protein digestibility (51–53).

Our results suggest that pullets and laying hens have a high tolerance to dietary fiber, without negatively affecting their protein digestion. This means that not only SFM but also probably other high-fiber-containing industrial by-products can be used at higher inclusion rates in the pullet and layer diets. The difference between the results of pullets and layers could be due to the digestive tract of the younger birds, similarly to broiler chickens, being more adaptive than that of the 50-week-old animals. It is known that the trypsin activity of the small intestine increases as the bird gets older (54). Very likely, the enzyme secretion of hens is higher than that of the restricted-fed pullets. Therefore, stimulating the gizzard motility by SFM and pancreatic enzyme secretion (55) was visible only with pullets. The reason for the impaired digestion of the two-branch chain amino acid is unknown. The investigations of the age effects on AA digestion are of specific interest because, in diet formulations, the same global digestibility values are used for all poultry species and age groups. Of course, this practice could cause inaccuracies.

Knowledge of the digestibility of amino acids is important in diet formulations because AA digestibility can vary greatly among different feedstuffs and among samples of the same ingredient (56). Currently, the use of ileal AA digestibility values is common in poultry and pig diet formulation. The so-called standardized ileal digestibility (SID) of amino acids means digestibility calculations based on the AA content of the ileum or terminal part of it. The standardization means the correction of the apparent digestibility with the basal endogenous amino acid losses (BEAAL) (25). Measuring the non-digested AAs from the ileum is more accurate since the AA content of the excreta is partly modified by the microbes in the ceca. The corrections with the endogenous amino acid losses (EAAL) are also important because the AA originated from the mucus, digestive enzymes, or other gut secretions also containing AAs (57). The advantage of the regression model used in this trial is that no additional measurement of EAAL is needed (26). This statement is, however, not entirely true since a part of the ileal EAAL does not belong to the BEAAL but is diet-specific. It is well known that the fiber content and the presence of anti-nutritive factors can also modify the amount of EAAL. This is the reason why, in the regression equations of this trial, the constants were not only positive. The most abundant amino acids in the ileal endogenous protein of poultry were glutamic acid, aspartic acid, threonine, proline, serine, and glycine. These amino acids are found in high concentrations in the intestinal and pancreatic secretions and mucoproteins, confirming that these are the major components of endogenous protein (57).

Comparing our results with the table’s amino acid digestibility values, the largest differences were observed in the digestibility of lysine (75–88%), threonine (70–85%), and histidine (77–88%). The reason for these big differences is partly that the table values are based on different methodologies. The values of NRC originate from the so-called precision feeding method, using adult cecectomised roosters, calculating the digestion from the excreta, and using EAAL corrections with N-free diets (58, 59). The EVONIK and CVB data are based mainly on *ad libitum*-fed broiler chickens and ileal samplings. The AA digestibility of feedstuffs has been calculated in this case using the difference method after incorporating the test feedstuff into a basal diet. In these methods, the inclusion rate could contribute to inaccuracy, since a low percentage increases the standard deviation of the determination and a high inclusion rate can already be depressive. The differences are also due to the animals. Using laying hens or pullets in these trials is rare because of the high price of the birds. Of course, the digestion potential of adult roosters, broiler chickens, laying hens, and restricted-fed pullets is different (60–63).

## 5 Conclusion

Sunflower meal is a locally available potential alternative to soybean meal in several countries. According to the results of this experiment, poultry can tolerate the higher structural fiber of SFM. Feeding sunflower meal at even 30% does not have a negative effect on the amino acid digestibility of the compound feeds. In the case of young pullets, the digestibility of several amino acids was even increased as a response to SFM inclusion. This result attracts attention to the importance of having age and species-specific AA digestibility coefficients for the more fibrous feedstuffs. There is high variance in the AA digestibility between the measured and table values of SFM’s amino acids. The main reason for this is the difference in the animal models of digestibility determinations.

## Data availability statement

The raw data supporting the conclusions of this article will be made available by the authors, without undue reservation.

## Ethics statement

The animal study was approved by the Institutional Ethics Committee (Animal Welfare Committee, Georgikon Campus, Hungarian University of Agriculture and Life Sciences). The study was conducted in accordance with the local legislation and institutional requirements.

## Author contributions

NS: Formal analysis, Investigation, Writing – original draft. ÁM: Investigation, Resources, Writing – original draft. KT: Investigation, Writing – review & editing. LP: Investigation, Writing – review & editing. BH: Investigation, Writing – review & editing. JP: Data curation, Writing – review & editing. KD: Conceptualization, Funding acquisition, Investigation, Resources, Supervision, Writing – original draft, Writing – review & editing.
